# Childhood Asthma and Environmental Interventions

**DOI:** 10.1289/ehp.8989

**Published:** 2007-01-25

**Authors:** Felicia Wu, Tim K. Takaro

**Affiliations:** 1 Department of Environmental and Occupational Health, University of Pittsburgh, Pittsburgh, Pennsylvania, USA; 2 Faculty of Health Sciences, Simon Fraser University, Burnaby, British Columbia, Canada

**Keywords:** childhood asthma, economic impacts, indoor air quality, indoor environments, public health interventions

## Abstract

**Background:**

Contaminants encountered in many households, such as environmental tobacco smoke, house dust mite, cockroach, cat and dog dander, and mold, are risk factors in asthma. Young children are a particularly vulnerable subpopulation for environmentally mediated asthma, and the economic burden associated with this disease is substantial. Certain mechanical interventions are effective both in reducing allergen loads in the home and in improving asthmatic children’s respiratory health.

**Results:**

Combinations of interventions including the use of dust mite-impermeable bedding covers, improved cleaning practices, high-efficiency particulate air vacuum cleaners, mechanical ventilation, and parental education are associated with both asthma trigger reduction and improved health outcomes for asthmatic children. Compared with valuated health benefits, these combinations of interventions have proven cost effective in studies that have employed them. Education alone has not proven effective in changing parental behaviors such as smoking in the home.

**Conclusions:**

Future research should focus on improving the effectiveness of education on home asthma triggers, and understanding long-term children’s health effects of the interventions that have proven effective in reducing asthma triggers.

People in modern societies spend the vast majority of their time—approximately 90%—in indoor environments, including homes, workplaces, schools, and public spaces such as restaurants and malls. Roughly 66% of that indoor time is spent in homes (Leech et al. 2002). Hence, indoor environmental quality in the home has a significant impact on public health and well-being. Indeed, indoor pollution has been ranked by both the U.S. Environmental Protection Agency (EPA) Science Advisory Board and the Centers for Disease Control and Prevention as a high environmental risk (Leung et al. 1997).

Although globally the greatest health risks are associated with particulate pollution from indoor biomass burning that kills an estimated 1.6 million people per year (World Health Organization 2002), the indoor environmental risks that are the focus of this article are related specifically to indoor air quality (IAQ) in higher-income countries. In this setting, indoor chemical contaminants include environmental tobacco smoke (ETS), nitrogen dioxide from space heaters and poorly ventilated furnaces, carbon monoxide, volatile organic compounds (VOCs), phthalates, and pesticides. Biological contaminants include antigens from house dust mites, molds, rodents, cockroaches, and animal dander. Dampness and endotoxins have also been implicated in health risks associated with indoor environments [Institute of Medicine (IOM) 2000, 2004; Thorne 2005]. Indoor air pollutants in the home may lead to the development and/or exacerbation of a variety of diseases and symptoms. Some known and postulated adverse health effects associated with poor indoor air quality are allergies, asthma, infection, hypersensitivity pneumonitis, inhalation fevers, mucosal irritation, central nervous system effects, psychologic effects (including depression), dermatitis, and even some forms of cancer (IOM 2000, 2004).

Asthma and allergic conditions in particular are believed to be associated primarily with exposure to contaminants common in indoor rather than outdoor environments (IOM 2000). The IOM has concluded there is sufficient evidence of a causal relationship between asthma development and exposure to house dust mite (IOM 2000). Substantial evidence indicates that children exposed to indoor air mold in the first years of their lives have a significantly higher probability of developing asthma (Jaakkola et al. 2005). There is sufficient evidence of a causal relationship between asthma exacerbation and exposure to cats, cockroaches, house dust mite, mold, and ETS in preschool-age children (IOM 2000). There is also increasing evidence that pollutants from vehicle traffic infiltrates indoors, adding to the risk of asthma and exacerbations (McConnell et al. 2006).

The number of self-reported asthma cases in the United States increased by 75% between 1980 and 1994 (Mannino et al. 1998). The most dramatic increase—160%—was seen in children younger than 4 years. From 1975 to 1994, the number of office visits for asthma increased from 4.6 million to 10.4 million. Almost 15 million Americans have asthma today (IOM 2000).

From a health–economics standpoint, the loss of quality of life and productivity from an early diagnosis of a lifelong chronic disease such as asthma is enormous—both to the individual and to the society. Moreover, when a child becomes sick, it is often the case that both a school day and a parent’s workday are lost. Thus, educational and productivity losses because of adverse health effects from indoor contaminants could be substantial. Individuals with low incomes, particularly in inner cities, are more likely to be living in substandard housing with severe structural problems, with moisture intrusion, poor ventilation, and associated mold and pest-related problems. These families are the least likely to have the means (money and education) by which to remediate such problems (Evans and Kantrowitz 2002; IOM 2004). Often, they also lack access to information regarding the extent of health problems associated with indoor asthma hazards and appropriate remediation responses. Therefore, the group most likely to suffer from indoor environment-induced asthma is children in low-income urban families.

Fortunately, many of the interventions to reduce asthma triggers in home environments are relatively simple. Recently, a 7-year follow-up of a Canadian birth cohort has confirmed previous suggestions that simple environmental interventions directed at the hazards noted above can prevent asthma in high-risk children (Arshad 2003; Chan-Yeung 2005). Intervention measures such as encasing mattresses and pillows with dust mite–impermeable cases, removing carpets, and more frequent cleaning of clothes, floors, and upholstered furniture can reduce exposure of families to potentially harmful contaminants in home environments. In this article we describe costs associated with asthma and suboptimal indoor environments in the United States, evaluate studies that have investigated links between home environmental interventions and reduction of asthma symptoms in children, and address the economic impacts of these interventions and the subsequent health outcomes.

## Costs of Asthma

There are heavy economic burdens associated with asthma. In addition to direct medical costs, the symptoms experienced by asthmatics lead to reduced productivity in the workplace and absenteeism from school and from work. There are also unaccounted costs of pain, suffering, and inconvenience associated with the disease. In 1994, asthma was estimated to cost U.S. society $13.7 billion [all monetary values, except where noted, in the article are given in 2005 U.S. dollars (USD)], through medical costs and the high number of lost workdays (Weiss et al. 2000). Fisk (2000) estimated that asthma, allergic rhinitis, and other associated airway allergic diseases cost $23 billion in terms of health care and indirect costs including lost work and lost school days.

There have been several attempts to valuate the economic impact of asthma that can be directly attributed to unhealthy indoor environments. Landrigan et al. (2002) judged that the environmentally attributable factor (EAF) in children’s asthma was 30%, with a range of 10–35%. At this EAF value, they estimated the total annual costs from U.S. children’s asthma caused by environmental exposures at $2.3 billion. As most environmentally mediated asthma is attributed to indoor exposures, most of this cost is likely because of unhealthy indoor environments. Nguyen et al. (1998) estimated the annual cost of asthma linked with dampness in residential buildings in Finland at $9.40 per person, based on direct medical costs and productivity losses. The corresponding per capita cost associated specifically with mold in buildings was $4.96.

In addition to direct medical costs and productivity losses, it is important to consider losses that result from pain and suffering from asthma. Contingent valuation studies that assessed people’s willingness to pay (WTP) to reduce respiratory symptoms found that individuals were willing to pay between $7 and $341 per additional respiratory symptom day avoided in a year (Berger et al. 1987; Loehman 1979). In another study, asthmatic respondents showed a WTP of an average of $61 for a 1-day reduction in bad-asthma days in a year (Rowe and Chestnut 1985).

Table 1 summarizes the literature on costs associated with dampness and related health symptoms, and the benefits from relieving symptoms. Although there is uncertainty and variability associated with these estimates, partly due to the differences in adverse effects being measured, these data show that the societal costs of indoor dampness and other indoor asthma hazards and the related respiratory illnesses are very large. The overall annual cost of asthma in the United States is estimated in the tens of billions USD, and the costs for asthmatic children specifically was about $2.3 billion in 2002 (Landrigan et al. 2002). In addition, the willingness-to-pay (WTP) to reduce bad-asthma days among asthma sufferers is substantial on a national scale.

## Home Environmental Interventions to Reduce Asthma Triggers

Many studies have assessed the effectiveness of individual or comprehensive strategies to reduce asthma triggers in homes, with subsequent improvement in children’s health. Of 32 studies published from 1992 to 2005, 24 reported randomized clinical trials of interventions in homes. Such interventions included increased mechanical ventilation, addition of bedding covers, vacuuming and cleaning, pest control methods, education programs to encourage parents not to smoke inside the home, and various combinations of the above interventions. Several articles had specific targets for allergen removal, such as ETS, cockroach, house dust mite, pet dander, and mouse allergens, whereas other articles described removal of combinations of potential home allergens. Fourteen studies measured whether children’s respiratory health specifically improved as a result of interventions to remove asthma triggers in the home (e.g., Krieger et al. 2005; Morgan et al. 2004). These studies fall into three categories: those that focused on one or more mechanical methods to reduce home environmental triggers, those that focused on education of asthmatic children and their parents, and those that used a combination of interventions incorporating both of the above. Table 2 summarizes the studies that tested various interventions in home environments to reduce asthma triggers and improve children’s health. The intervention type (“mechanical” = mechanical home-based environmental intervention to reduce asthma triggers; “education” = educating parents and/or asthmatic children; and “combination” = a suite of interventions including both mechanical interventions and education) is listed, as well as a brief description of the study and any significant environmental and health effects reported.

### Mechanical methods to reduce home environmental asthma triggers

In this present article we discuss four categories of mechanical methods to reduce asthma triggers in the home: bedding covers, vacuum cleaners, improved ventilation, and heating. Two projects evaluated the effects of mite-impermeable mattress and pillow covers on dust mite levels and children’s respiratory health (Brunekreef et al. 2002; Halken 2004). In both these studies, levels of house dust mites in the bedrooms were significantly reduced. However, the results of the studies on changes in children’s respiratory health are mixed. While Halken (2004) found that semipermeable mattress and pillow encasings significantly reduced house dust mite exposure and the need for inhaled steroids among asthmatic children diagnosed with allergies to house dust mite, Brunekreef et al. (2002) showed no important clinical benefits in children up to 2 years of age, whose mothers had house dust mite allergy, from the mite-impermeable bedding covers despite a significant reduction in mite-allergen levels in the intervention homes. There may be several reasons for this discrepancy in findings. Brunekreef et al. (2002) evaluated respiratory health in children up to 2 years of age, which is earlier than most children exhibit asthma. Inhaled steroids use was not described. Also, children whose mothers have dust mite allergy may not have the allergy themselves at such an early age and hence would not have shown an improvement in respiratory function if house dust mite counts were reduced.

High-efficiency particulate air (HEPA) vacuum cleaners versus standard vacuum cleaners were tested for effectiveness in removing allergens and improving respiratory health in asthmatic children (Popplewell et al. 2000). It was found that HEPA vacuum cleaners significantly reduced house dust mite and cat and dog allergens throughout the home after 12 months of use, whereas the standard cleaners reduced cat allergens only in mattress dust samples. Clinically, house dust mite–allergic patients in the HEPA group showed improvements in peak respiratory flow rate and bronchodilator use after 12 months.

HEPA vacuum cleaners were also tested in conjunction with mechanical ventilation (Warner et al. 2000) to reduce house dust mites. Specifically, this study tested the efficacy of a whole-house mechanical ventilation system with heat recovery (MVHR) unit; each unit consisted of a heat exchanger and two fans with a manually operated boost switch for the bathroom and a filter on the air supply. It was found that homes with MVHR units achieved significantly lower humidity levels than those without, with an associated reduction of house dust mite counts. Histamine levels in asthmatic patients improved. The addition of HEPA vacuuming further reduced dust mite concentrations in homes; however, it did not have a significant additional impact on health improvements.

Another ventilation study measured the impact of bedroom and living room air cleaners on asthmatic children’s symptoms (van der Heide 1999). It was found that after 3 months of intervention with active air cleaners, substantial amounts of airborne cat and dog allergens were captured by the cleaners, and airway hyperresponsiveness in the asthmatic children decreased significantly.

Central heating as a prophylactic to indoor dampness and corresponding children’s asthma symptoms was the focus of another project (Somerville et al. 2000). The installation of central heating in several homes was associated with significantly reduced dampness and improved energy efficiency. Children’s adverse respiratory symptoms such as nocturnal cough were significantly reduced, and school-age children lost less time from school for asthma.

In total, these mechanical interventions to improve home environments have largely proven successful in reducing asthma triggers—house dust mite, cat and dog allergens, and dampness—significantly in the home. In many cases, this has led to documented improvements in children’s respiratory health.

### Education of asthmatic children and their parents on healthier home environments

Several studies have focused on education of parents and children regarding a particular intervention. Two studies (McIntosh et al. 1994; Wakefield et al. 2002) tested the efficacy of educating parents not to smoke in homes to reduce children’s exposure to a key asthma exacerbator. Fitzpatrick et al. (1992) tested the efficacy of a weeklong asthma camp for asthmatic children and their parents to decrease hospital visits and sick days from school.

Educating asthmatic children and their parents has had mixed results. The weeklong asthma camp intervention to educate children and their families resulted in clinically significant reductions in school absences, emergency department visits, and hospitalizations (Fitzpatrick et al. 1992). However, Wakefield et al. (2002) and McIntosh et al. (1994) found no significant change in parental home-smoking behaviors or in the health of asthmatic children as a result of educational programs encouraging parents to cease smoking in the home.

### Combination of mechanical and educational methods to remove home asthma triggers

Three series of studies used a combination of interventions, including education and various means of physical remediation, to control asthma triggers in home environments. The Seattle–King County Healthy Homes Project, which is summarized by Krieger et al. (2005), employed a community health worker intervention to decrease exposure to indoor asthma triggers in low-income households with asthmatic children. Community health workers provided indoor environmental assessments, education and support for behavior change, and resources to control triggers. Participants were randomly assigned to a high-intensity group receiving a mean of seven home visits and full set of resources or to a low-intensity group receiving a single visit and limited resources. This study demonstrated both significant allergen count reductions (measurements of condensation, roaches, moisture, cleaning behavior, dust weight, dust mite antigen, and total antigens) and improved children’s health (reduced symptom days and urgent care visits) as a result of high-intensity interventions (Krieger et al. 2005). The higher-intensity group improved more than the lower-intensity group in its Pediatric Asthma Caregiver Quality of Life score (*p* =0.005) and asthma-related urgent health services utilization (*p* =0.026) but not asthma symptom days, after adjustment for baseline differences. Participant actions to reduce asthma triggers increased in the higher-intensity group but not in the lower. The higher intensity group showed improvement in measurements of condensation, roaches, moisture, dust weight, dust mite antigen, and total antigens above a clinical effect cut-point, effects not demonstrated in the lower intensity group.

Two multicenter randomized-controlled home intervention trials have been conducted in other inner city U.S. populations, The first, the National Cooperative Inner-City Asthma Study (NCICAS) used a social worker–implemented home intervention (Evans et al. 1999), whereas the second used two “environmental counselors” to deliver the home intervention (Morgan et al. 2004). The inner-city home environmental interventions were specifically targeted to the allergies of the individuals living in the home and the evidence of exposure that occurred in the home. The environmental interventions targeted the common allergens of dust mite, cockroach, pet dander, rodents, ETS, and/or mold, and included allergen-impermeable bedding covers, air purifiers with HEPA filters, HEPA vacuum cleaners, professional pest control, and an educational component. These interventions led to significantly lower allergen loads in the home, fewer symptom days in the asthmatic children, and reduced albuterol inhaler use and fewer unscheduled clinic visits.

Two studies (Carter et al. 2001; Hayden et al. 1997) described a project in which asthmatic children in Atlanta, Georgia, were randomized into three groups: two groups with home visits by health professionals, one with active avoidance (including impermeable bedding covers, hot washing of bedding, and cockroach bait) and one with placebo avoidance (permeable bedding covers and cold washing of bedding), and a control group with no home visits. The combination of bedding covers, hot water washes of bedding, and removal of carpets resulted in improved respiratory function even among asthmatic children admitted to the hospital (Hayden et al. 1997). Interestingly, when both actual and placebo interventions were employed for allergen reduction in the home (Carter et al. 2001), children’s hospitalization rates for asthma dropped in both the actual and placebo intervention groups, but no significant difference in hospitalization was observed between the two groups. As in many of these intervention studies, it was concluded that the home visitation itself influenced asthma management among families.

## Economic Impacts of Home Environmental Interventions

Few studies have attempted to assess the cost-effectiveness of various home environmental interventions to reduce children’s asthma symptoms. Limited data are available concerning how much can be accomplished through environmental interventions. Fisk (2000) estimates that improving indoor environments can result in as much as a 10–30% reduction in asthma symptoms and the associated costs. This would translate to an annual savings in the United States of $2 billion to $4 billion. However, this includes not just homes but also office buildings and pertains to both children and adults.

Our analysis described above indicates that certain types of interventions are effective in reducing home environmental allergens and improving asthmatic children’s health. The question remaining is how affordable are they, given that many asthmatic children in the United States live in low-income urban households. Allergen-impermeable bedding covers have proven effective in reducing allergen exposure; a complete set (for pillows, mattresses, comforters, and box springs) can cost about $150–$450 [for a twin-size to king-size bed; the website http://www.allergycontrol.com gives sample costs for these and other interventions (Allergy Control Products 2006)]. HEPA vacuum cleaners, which have also proven effective in allergen removal and improved children’s health, cost about $200–$1,000. HEPA air cleaners for a single room range from $100 to $500, whereas single-room dehumidifiers range from $200 to $800. Other more intensive interventions such as installation of a whole-house ventilation system or a central heating system are likely to be more expensive, although they too have proven effective in improving asthmatic children’s health. Educational programs have an enormous range of costs, and applied alone have not consistently proven effective in long-term changes in parental behavior.

Cost data are available from three of the comprehensive intervention projects: the Seattle–King County Healthy Homes project (Krieger et al. 2005), the National Cooperative Inner-City Asthma Study (NCICAS; Sullivan et al. 2002), and Inner-City Asthma Study groups (ICAS; Kattan et al. 2005; Morgan et al. 2004). Krieger et al. (2005) made certain assumptions about the costs of hospital admissions, emergency department visits, and clinic visits, and compared these costs with benefits seen in the asthmatic children that participated in their study in terms of reduced number of visits. Within the high-intensity group, the estimated net decrease in 2-month urgent care costs between baseline and exit ranged from $22,084 to $36,700 ($201–$334 per child), and within the lower-intensity group, from $19,246 to $32,756 ($185–$315 per child). Although this study did not collect follow-up data on both groups, a 6-month follow-up of a randomized sample of the higher-intensity group demonstrated that urgent health care use continued to decline after the active intervention ceased.

Both multicenter inner-city 1-year interventions were also cost effective at reducing morbidity in inner-city children with atopic asthma. Cost-effectiveness analyses on both morbidity and atopic asthma have been conducted by Sullivan et al. (2002) and Kattan et al. (2005). Both interventions were cost effective based-upon symptom-free days (SFD) The NCICAS intervention cost was $9.20 per SFD [95% confidence interval (CI), $12.56–$55.29]. The ICAS estimated the costs of the tailored, home-based environmental intervention at $1,469 per family (Kattan et al. 2005). The children who received this intervention had 19% fewer unscheduled clinic visits, a 13% reduction in the use of albuterol inhalers, and 38 more symptom-free days over the 2-year course of the study than those in the control group. This translated into an estimated intervention cost of $27.57 per symptom-free day (95% CI, $7.46–$67.42). Because 1-day of symptoms for an asthmatic child could include an unscheduled clinic visit ($49.34), an emergency department visit ($390), or an inpatient hospital day ($1,134), this type of intervention is indeed cost effective (Kattan et al. 2005).

## Discussion

Indoor environmental quality in homes is an important health concern, particularly for infants and small children who are more susceptible to the adverse health effects that can result from exposures to hazards encountered in homes. In particular, pediatric asthma, a disease that has been increasing dramatically over the last three decades, is associated with pollutants found indoors. The medical and societal costs of asthma are significant. These costs are multiplied when lifetime medical and productivity and other less direct costs are considered. Because children can develop asthma from allergens encountered indoors and these allergens exacerbate existing asthma, home environmental interventions are important from a health standpoint as well as economically.

Simple mechanical home interventions are effective in the reduction of allergen loads in the home, in reducing symptoms and urgent care associated with asthma, and in preventing the emergence of the disease. Use of bedding covers, HEPA vacuum cleaners and air cleaners, increased ventilation, and central heating in the home can reduce indoor air allergens and often improve children’s respiratory health. A combination of interventions, involving both mechanical methods for allergen reduction and educational efforts of asthmatic children and their parents, has proven effective in asthma prevention and trigger reduction and has improved health outcomes for asthmatic children.

Education and information dissemination alone have generally not proven effective in reducing indoor asthma triggers and the resulting asthma symptoms. Pilot projects in which educational interventions encouraged parents not to smoke in the home showed no significant effect on parental smoking behaviors, indicating that for this addictive behavior more effort is necessary to deliver effective messages. Even when parents are able to identify asthma triggers in their child’s environments, they may not always know proper interventions to control the triggers. In a nationwide survey, Cabana et al. (2004) found that although 80% of parents of asthmatic children were able to identify at least one environmental asthma trigger, and although 82% of these parents attempted to control such triggers, less than half the control actions were thought by the authors to be useful. Hence, better education for parents about effective control methods for home asthma triggers is needed. Clark and Valerio (2003) and Clark and Partridge (2002) provide guidelines for improving patient and parental education on asthma and describe the potential usefulness of health behavior theories and improving the educational role and skills of clinicians.

The cost of effective interventions is important to consider as well as the extent to which an asthmatic child’s parent is able to achieve the home interventions independently without external aid. It is important to remember that children’s asthma disproportionately affects low-income urban households (IOM 2000) in which parents may find it difficult to achieve some of the more costly or complicated interventions described in the studies above. Typically, the costs for bedding covers, HEPA vacuum cleaners and air cleaners, and dehumidifiers range from $100 to several hundred dollars. These types of interventions can be useful for removing multiple allergens. In the few studies that have evaluated the economic impacts of a combination of home environmental interventions to reduce children’s asthma symptoms, the interventions have proven cost effective in terms of improved respiratory health outcomes. This indicates that investments in providing low-cost interventions and educating the public about improving their home environments are likely to reap significant and lasting benefits for asthmatic children and prevent asthma in families with children at risk.

Future work may focus on understanding long-term effects of the interventions that have proven effective in reducing home environmental asthma triggers and improving symptoms and quality of life in children with asthma. Improvements in educational design may be needed to motivate parents to change behaviors such as smoking and more frequent cleaning in the home. It is important to consider potential risks of synthetic bedding, plasticizers, new paint, and other indoor emissions as subjects for future intervention research on children’s asthma. Also, future research could ascertain whether such forms of intervention and education are effective in reducing the adverse effects of other environmentally mediated diseases from hazards inside the home and how genes and these environmental exposures interact.

## Figures and Tables

**Table 1:** Costs associated with respiratory illnesses attributable to poor indoor environments and the benefits from relieving symptoms.

Cost or benefit to society	Value (2005 USD)	Reference
Medical and productivity costs of asthma across U.S. population	$13.7 billion	Weiss et al. 2000
Costs in United States of asthma, allergic rhinitis, and other associated allergic airway diseases	$23 billion	Fisk 2000
Cost in United States of environmentally attributable children’s asthma	$2.3 billion	Landrigan et al. 2002
Direct and indirect annual costs per capita in Finland of asthma due to damp residences	$9.40	Nguyen et al. 1998
Direct and indirect annual costs per capita in Finland of asthma due to mold	$4.96	Nguyen et al. 1998
WTP in the United States per additional respiratory symptom day avoided per year (per capita)	$7–$341	Berger et al. 1987, Loehman 1979
WTP in the United States per additional bad asthma day avoided per year (per capita)	$61	Rowe and Chestnut 1985

**Table 2:** Asthma intervenion trials.

Study	Intervention type	Description of intervention	Home environmental effects	Health effects
Brunekreef et al. 2002	Mechanical	Mite-impermeable bedding covers	Lower house dust mite count	No important clinical benefits in children < 2 years of age
Halken 2004	Mechanical	Mite-impermeable bedding covers	Lower house dust mite count	Reduced need for inhaled steroids in asthmatic children
Popplewell et al. 2000	Mechanical	HEPA and standard vacuum cleaners	Reduced house dust mite, cat and dog allergens with HEPA	Improved peak respiratory flow rate, bronchodilator usage
Warner et al. 2002	Mechanical	HEPA vacuum cleaners and whole-house mechanical ventilation system	Reduced dampness and house dust mite count	Improved histamine levels with whole-house ventilation; no significant added benefit from HEPA
van der Heide 1999	Mechanical	Bedroom and living room air cleaners	Reduced airborne cat and dog allergen	Improved peak expiratory flow rate; reduced airway hyper responsiveness
Somerville et al. 2000	Mechanical	Central heating systems	Reduced dampness; improved energy efficiency	Improved asthma outcome measures; fewer sick school days
Wakefield et al. 2002	Education	Parents refraining from smoking in the home	No significant difference in parental behavior	No significant improvement in children’s health
McIntosh et al. 1994	Education	Parents refraining from smoking in the home	No significant difference in parental behavior	No significant improvement in children’s health
Fitzpatrick et al. 1992	Education	Asthma camp for asthmatic children and their parents	Improved use of medication and breathing exercises	Reduced school absences, emergency department visits, and hospitalizations
Krieger et al. 2005	Combination	Community health workers deployed to Seattle, WA, homes: high-intensity, low-intensity interventions	Reduction in numerous home allergens	Reduced children’s asthma symptom days and use of urgent health services
NCICAS Kattan et al. 2005 Morgan et al. 2004 Sullivan et al. 2002	Combination	Seven cities: comprehensive home environmental interventions and education targeted to children’s allergies	Reduction in numerous home allergens	Reduction in asthma symptom days in children, use of albuterol inhalers, and unscheduled clinic visits
Carter et al. 2001 Hayden et al. 1997	Combination	Interventions in homes of hospitalized asthmatic children with home visits: active, placebo, and control	Reduction in house dust mite	Reduced children’s acute asthma hospital visits; no difference in active vs. placebo groups
